# Infusion‐Site MSSA as the Source of Confirmed Toxic Shock Syndrome in a 9‐Year‐Old on Automated Insulin Delivery System: A Case Report

**DOI:** 10.1155/crpe/5429201

**Published:** 2026-08-10

**Authors:** Fabio Rotondo, Sofia Siena, Carmela De Meco, Pasquale Maccarone, Morena Mansueto, Maria Rosa Pastore, Irene Rutigliano

**Affiliations:** ^1^ Department of Pediatrics, IRCCS “Casa Sollievo Della Sofferenza”, San Giovanni Rotondo, Italy, operapadrepio.it; ^2^ Department of Pediatrics, University of Foggia, Foggia, Italy, unifg.it

**Keywords:** AID, automated insulin delivery, diabetes mellitus, pediatric infection, toxic shock syndrome, TSS

## Abstract

Staphylococcal toxic shock syndrome is a rare toxin‐mediated illness. A 9‐year‐old girl on automated insulin delivery presented with shock, multiorgan dysfunction, rash, and gastrointestinal symptoms. An infected infusion site grew methicillin‐susceptible *Staphylococcus aureus*. She met CDC criteria for TSS, highlighting infusion sites as sepsis foci and supporting continuation of automated insulin delivery during critical illness.

## 1. Background

Type 1 diabetes mellitus (T1DM) is a chronic autoimmune disease characterized by beta‐cell destruction and absolute insulin deficiency, requiring lifelong exogenous insulin from diagnosis. In children and adolescents, modern management combines intensive insulin therapy with structured education to optimize glycemic outcomes and safety [1–3]. Multiple daily injections (MDI) using basal–bolus regimens remain standard care, while continuous subcutaneous insulin infusion (CSII) and, more recently, automated insulin delivery (AID) systems have emerged as attractive alternatives [1–4]. By integrating continuous glucose monitoring with algorithm‐driven insulin adjustments, AID systems can increase time in range and reduce both hypoglycemia and hyperglycemia, and contemporary guidelines recommend early consideration of pump therapy [1–4]. Systematic reviews and pediatric cohort studies further support improvements in glycemic control and treatment satisfaction with CSII and AID compared with MDI [4–6]. However, pump therapy shifts risk from injection‐related issues to device‐ and infusion‐set‐related vulnerabilities. Rapid interruption of insulin delivery (e.g., tubing kinks, cannula occlusions, and empty reservoirs) can precipitate hyperglycemia and diabetic ketoacidosis (DKA) within hours because pumps use rapid‐acting insulin only [1, 4]. Infusion‐site complications, including erythema, induration, and abscess, are relatively common; while most are localized, invasive infections may occur, underscoring the need for vigilant site inspection and prompt, source‐directed therapy when systemic illness is suspected [7–9]. Acute complications linked to infusion‐set failure and site infections, reinforce the importance of standardized site‐change protocols, skin antisepsis, and patient/family training [9, 10]. In practice, safe and effective AID system use in pediatrics hinges on comprehensive education (including sick‐day rules), routine rotation of sites, early recognition of delivery failure, and rapid escalation of care when sepsis or DKA is a concern [1–3].

## 2. Case Report

**FIGURE 1:**
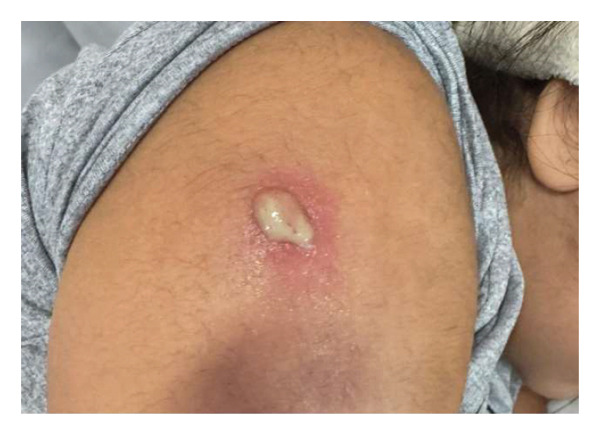
Purulent drainage at CSII site.

A prepubescent, 9‐year‐7‐month‐old girl with T1DM and autoimmune thyroiditis, treated with hybrid closed loop AID system and levothyroxine (0.3 mcg/kg/die), developed vomiting and nonbloody diarrhea, followed by fever up to 39°C, and progressive obtundation. She arrived at the emergency department drowsy but reactive subsequently developed hypotension (70/30 mmHg‐< 5^th^ percentile for sex and age) and a Glasgow Coma Scale score of 12/15. Her mother reported a malfunction of the insulin pump, localized on the right shoulder. Inspection of the area revealed only mild erythematous swelling. Capillary glucose was 200 mg/dL and capillary ketonemia 0.5 mEq/L; thus, DKA was excluded. Initial venous blood gas showed pH 7.28 and lactate 7.4 mmol/L. Urgent laboratory tests revealed a white blood count of 17,310/µL (95.5% neutrophils), hemoglobin 12.5 g/dL, platelets 176,000/µL; blood urea nitrogen 63 mg/dL, creatinine 1.7 mg/dL; AST 191 U/L, ALT 189 U/L; INR 1.81, aPTT 38 s (ratio 1.46); CRP 10.6 mg/dL; procalcitonin > 100 µg/L. Given this presentation, consistent with septic shock, blood cultures were obtained, and she was admitted to the intensive care unit (ICU). Shortly after admission, she developed a diffuse erythematous rash that resolved spontaneously within approximately 15 min. Empirical antimicrobial therapy with prolonged infusion of meropenem 1 g every 8 h and teicoplanin loading (400 mg IV twice, then 400 mg IV daily) which was discontinued after one dose, due to an erythematous eruption. Brief vasoactive support with dopamine was needed. Glycemic control was consistently within target with the AID system, maintaining an average time in range (70 mg‐180 mg/dL) of 83%, time below range 2%, time above range 15%, over the first four days, despite ongoing shock and intensive care needs. The patient showed a rapid response to antibiotic therapy and, after four days in the ICU, was transferred to the pediatric ward. During local wound care of the erythematous area on the right shoulder, purulent drainage was observed (Figure 1), and a swab was obtained for culture. She completed IV meropenem therapy and then transitioned to oral amoxicillin‐clavulanate 1 g twice daily guided by the MSSA susceptibility profile obtained from the culture. Blood cultures remained negative.

**FIGURE 2:**
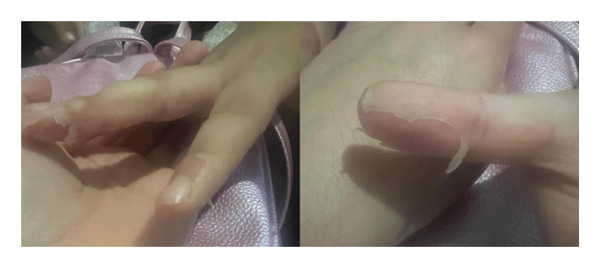
Acral hands peeling observed a few days postdischarge.

Local wound care (saline compresses and antiseptic/topical antibiotic to the right arm) was performed with daily dressing changes. She remained afebrile with progressive clinical recovery and declining inflammatory markers. She was discharged with amoxicillin‐clavulanate therapy and topical fusidic acid. Caregivers were instructed to keep the affected area clean and dry, monitor for local signs of infection, avoid placing sensors or infusion sets over erythematous skin, and seek urgent reassessment for fever or new lesions. Outpatient follow‐up was scheduled for 6 days later. Peeling of the distal fingertips was noted a few days postdischarge, approximately 15 days after the onset of symptoms (Figure 2). The follow‐up evaluation, including abdominal ultrasound, laboratory testing, and urine studies, was unremarkable. The clinical presentation, laboratory results, transient generalized rash, documented *Staphylococcus aureus* infection at the infusion site, and subsequent acral desquamation were consistent with, and ultimately fulfilled, diagnostic criteria for staphylococcal toxic shock syndrome (TSS).

## 3. Discussion

In the post‐2000 era, staphylococcal TSS remains rare and broadly stable at roughly 0.03–0.07 cases per 100,000 persons per year in high‐income nations [11]. National surveillance from the UK estimates an average annual TSS incidence of 0.07/100,000, with nonmenstrual TSS now predominating and occurring at younger ages, findings directly relevant to pediatrics [12]. The pathogenesis of this case is consistent with toxin‐mediated staphylococcal disease rather than persistent bloodstream infection. *S. aureus* exotoxins (e.g., TSST‐1 and enterotoxins) act superantigens activating large numbers of T cells, resulting in massive cytokine surge, capillary leak and shock [13, 14]; a small, localized focus, such as an infected infusion site, can suffice as the toxin source [9, 14].

On admission, our young patient presented in critical condition, with reported insulin pump malfunction and nonspecific gastrointestinal and neurologic symptoms, raising concern for complications of Type 1 diabetes. We initially included DKA in the differential diagnosis, but it soon became unlikely given the absence of hyperglycemia and the low ketone level (0.5 mEq/L) with mild acidemia. Septic shock remained a concern because the patient had fever, marked hypotension, and multisystem involvement (gastrointestinal, renal, hepatic, and central nervous system). However, blood cultures were negative. The rapid onset of the symptoms and the clinical features as the documented erythematous rash, the abscess, and subsequent acral desquamation several days later supported the toxin‐mediated process rather than DKA or nonspecific septic shock. Taken together, these features establish the diagnosis of confirmed staphylococcal TSS according to the Centers for Disease Control 2011 surveillance criteria for nonstreptococcal TSS (Table 1) [13]. The initial antimicrobial strategy reflected the severity of presentation and the need for immediate broad‐spectrum coverage in a child with septic shock of unclear origin. However, once staphylococcal TSS became a diagnostic consideration, targeted antitoxin therapy with clindamycin would have aligned more closely with established recommendations. Postdischarge acral desquamation was self‐limited, classically seen during convalescence of staphylococcal TSS [7–11]. Comparable cases of TSS associated with insulin pump infusion sites have been described rarely. The earliest pediatric report from the Centers for Disease Control and Prevention (CDC) described a 12‐year‐old girl using CSII who developed a *Staphylococcus aureus* abscess at the infusion site complicated by TSS, with fever, hypotension, multiorgan involvement, and recovery after pump discontinuation and antistaphylococcal therapy [8]. Tanner et al. reported two young women on insulin pump therapy who developed staphylococcal TSS arising from infected infusion sites, with a similar constellation of gastrointestinal prodrome, shock, diffuse erythema, and delayed acral desquamation [7]. Larger series of pump users confirm that infusion‐site inflammation and infection are relatively frequent, whereas toxin‐mediated syndromes remain exceptional events, usually limited to isolated case reports [9–11].

**Table 1:** Diagnostic criteria of nonstreptococcal toxic shock syndrome (TSS).

Section	Criterion	Subcriterion	Description
Clinical criteria	Fever		Temperature ≥ 38.9°C (102.0°F)
	Rash		Diffuse macular erythroderma
	Desquamation		1 to 2 weeks after onset of rash
	Hypotension	For adults	Systolic blood pressure ≤ 90 mmHg
		For children < 16 years of age	Systolic blood pressure less than 5th percentile by age
	Multisystem involvement (3 or more of the following organ systems)	Gastrointestinal	Vomiting or diarrhea at onset of illness
		Muscular	Severe myalgia or creatine phosphokinase elevation > 2 times the upper limit of normal
		Mucous membranes	Vaginal, oropharyngeal, or conjunctival hyperemia
		Renal	Blood urea nitrogen or serum creatinine > 2 times the upper limit of normal or pyuria (> 5 leukocytes/high‐power field) in the absence of urinary tract infection
		Hepatic	Bilirubin or transaminases > 2 times the upper limit of normal
		Hematologic	Platelets < 100,000/microL
		Central nervous system	Disorientation or alterations in consciousness without focal neurologic signs when fever and hypotension are absent

Laboratory criteria	Cultures		Cultures (blood or cerebrospinal fluid) negative for alternative pathogens (blood cultures may be positive for Staphylococcus aureus)
	Serologic tests		Serologic tests negative (if obtained) for Rocky Mountain spotted fever, leptospirosis, or measles

Case classification	Probable case		A case which meets the laboratory criteria and 4 of the 5 clinical criteria
	Confirmed case		A case which meets the laboratory criteria and all 5 of the clinical criteria, including desquamation (unless the patient dies before desquamation occurs)

*Note:* UpToDate, as per 2011 Centers for Disease Control and Prevention (CDC) criteria.

From a metabolic perspective, children treated with MDI frequently struggle to maintain stable glycemia and recommended TIR targets, particularly during intercurrent illness or hospitalization. Large pediatric cohorts and systematic reviews show that youths managed with MDI or nonautomated pump therapy often spend well below the recommended 70% and higher of time within 70–180 mg/dL, with substantial hyperglycemic excursions and increased risk of DKA [1, 3–6, 15–18] Hybrid closed‐loop and other AID systems consistently improve TIR by roughly 10%–15% and modestly lower HbA1c without increasing hypoglycemia, in randomized trials and real‐world cohorts of children and adolescents, including those transitioning from MDI [5, 15–18]. In our patient, continuation of AID therapy during the first four ICU days yielded an average TIR of 83% despite shock, vasopressor support, and evolving multiorgan dysfunction and was associated with the absence of DKA or clinically significant hypoglycemia. These observations align with emerging clinical data showing that modern AID systems can safely maintain high TIR in children and adolescents during routine care and with early case reports of their use during hospitalization and minor surgery [15–19].

Limitations of this report include the absence of toxin assays. Strengths are the clear clinical and microbiological linkage to the infusion site, the documented generalized rash, and the prospective biomarker trajectory consistent with toxin‐mediated illness. For clinicians, the take‐home message is: in any child on an AID system presenting with shock or systemic illness, the infusion site should never be overlooked, and site and blood cultures should be obtained early. At the same time, our case suggests that, when appropriately supervised, continuing AID therapy during critical illness can help maintain stable glycemic control and prevent DKA, and that standardized site‐care protocols and education are essential to maximize the benefits of AID while minimizing infusion‐site‐related risks.

## Funding

No funding was received for this study.

Open access publishing was facilitated by the Universita degli Studi di Foggia, as part of the Wiley‐CRUI‐CARE agreement.

## Consent

Written informed consent was obtained from the patient’s parents/legal guardians for the publication of this case report and any accompanying images.

## Conflicts of Interest

The authors declare no conflicts of interest.

## Supporting Information

Additional supporting information can be found online in the Supporting Information section.

## Supporting information


**Supporting Information** This case report was prepared with reference to the CARE (CAse REport) guidelines.

## Data Availability

The data that support the findings of this study are available from the corresponding author upon reasonable request.
