# Supplementary material for: Infusion‐Site MSSA as the Source of Confirmed Toxic Shock Syndrome in a 9‐Year‐Old on Automated Insulin Delivery System: A Case Report
Source: Case Rep Pediatr. 2026 Aug 10;2026:5429201. doi: 10.1155/crpe/5429201 (PMC13455039; doi:10.1155/crpe/5429201)
Supplement: Supplementary file 1 [file CRPE-2026-5429201-s001.docx]

| **Checklist item description** | **Present in the manuscript (yes/no)** |
| --- | --- |
| 1. Title: Clearly reflects the case | YES |
| 2. Key Clinical Message: One clear take-home message, ≤50 words | NO |
| 3. Abstract (optional): Unstructured, no subheadings. | YES |
| 4. Keywords: 4-6 relevant keywords | YES |
| 5. Introduction: Briefly explain why the case is unique (include key references). | YES |
| 6. Patient Information    - De-identified patient details    - Main symptoms/concerns    - Medical, family, psychosocial, and genetic history (if relevant)    - Prior interventions and outcomes | YES |
| 7. Clinical Findings: Key physical exam and clinical findings. | YES |
| 8. Timeline: Chronological summary (table/figure encouraged). | YES |
| 9. Diagnostic Assessment    - Tests performed (labs, imaging, etc.)    - Diagnostic challenges    - Final diagnosis with differentials    - Prognosis (if relevant) | YES |
| 10. Therapeutic Intervention  - Type (medical, surgical, etc.) - Details (dose, duration) - Changes and rationale | YES |
| 11. Follow-up and Outcomes  - Clinical and patient-reported outcomes - Follow-up results - Adherence/tolerance - Adverse events | YES |
| 12. Discussion  - Strengths and limitations - Comparison with literature - Rationale for conclusions | YES |
| 13. Conclusion: Final take-home message (1 short paragraph, no references) | YES |
| 14. Ethics & Consent   - Confirm informed consent was obtained - Full patient anonymization along the manuscript | YES |
| 15. Length   - Case reports: 1,000-3,000 words - Case images: ≤500 words - Case videos: ≤100 words | YES |
| 16. References   - Case reports: no limit - Case images: ≤3 references - Case videos: ≤2 references - No specific format but use a consistent format throughout the manuscript | YES |
| 17. Case Series: Maximum of 3 cases | YES |
| 18. Letters to the Editor   - Written in response to a manuscript that has been published in Clinical Case Reports - ≤3000 words |  |
| 19. Figures   - High-quality images only - No photos of imaging screens - Ensure full patient anonymization | YES |
